# Enhancing *Monascus* Pellet Formation for Improved Secondary Metabolite Production

**DOI:** 10.3390/jof9111120

**Published:** 2023-11-19

**Authors:** Xizi Zhang, Huiqian Liu, Mengyao Zhang, Wei Chen, Chengtao Wang

**Affiliations:** Beijing Advanced Innovation Center for Food Nutrition and Human Health, Beijing Engineering and Technology Research Center of Food Additives, School of Food and Health, Beijing Technology and Business University, Beijing 100048, China; zhangxizi1999@163.com (X.Z.); q2223599235@163.com (H.L.); 2230201051@st.btbu.edu.cn (M.Z.)

**Keywords:** *Monascus*, pellet formation, secondary metabolites, fermentation, industrial applications

## Abstract

Filamentous fungi are well-known for their ability to form mycelial pellets during submerged cultures, a characteristic that has been extensively studied and applied. However, *Monascus*, a filamentous saprophytic fungus with a rich history of medicinal and culinary applications, has not been widely documented for pellet formation. This study aimed to investigate the factors influencing pellet formation in *Monascus* and their impact on citrinin production, a key secondary metabolite. Through systematic exploration, we identified pH and inoculum size as critical factors governing pellet formation. *Monascus* exhibited optimal pellet growth within the acidic pH range from 5 to 6, resulting in smaller, more homogeneous pellets with lower citrinin content. Additionally, we found that inoculum size played a vital role, with lower spore concentrations favoring the formation of small, uniformly distributed pellets. The choice of carbon and nitrogen sources also influenced pellet stability, with glucose, peptone, and fishmeal supporting stable pellet formation. Notably, citrinin content was closely linked to pellet diameter, with larger pellets exhibiting higher citrinin levels. Our findings shed light on optimizing *Monascus* pellet formation for enhanced citrinin production and provide valuable insights into the cultivation of this fungus for various industrial applications. Further research is warranted to elucidate the molecular mechanisms underlying these observations.

## 1. Introduction

**Figure 1:**
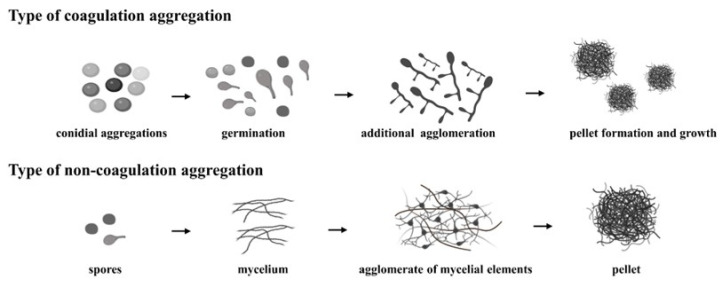
Description of the process of coagulative and non-coagulative types of pellets.

Filamentous fungi play a pivotal role in various industrial processes, contributing significantly to the production of enzymes, organic acids, antibiotics, and cholesterol-lowering agents through fermentation [1]. In submerged culture, these fungi exhibit two primary forms: mycelium aggregates forming pellets and uniformly dispersed suspended mycelium, fostering uniform growth [2]. The process of filamentous fungal pellet formation has been extensively studied, revealing two distinct typologies: coagulation and non-condensing, as illustrated in Figure 1. Coagulation-type pellets result from the coalescence of numerous spores during the pre-fermentation phase, followed by spore germination and subsequent mycelial tip growth. In contrast, non-condensing pellets undergo spore germination preceding pellet formation [3]. Fungi cultivated in pellet form offer several advantages, such as low fermentation broth viscosity, ease of biomass harvesting, and efficient oxygen diffusion [4].

The production of fungal products, whether target products or secondary metabolites, varies based on the morphological characteristics of the fungus [5]. Metabolites produced in pellet form typically exhibit higher yields, while fungi growing in suspended mycelia are more conducive to enzyme production [6]. However, the choice of form depends on the fungus species and external conditions. For example, *Aspergillus terreus*, characterized by small-diameter mycelial spheres, provides a favorable environment for lovastatin synthesis [7]. Conversely, *Aspergillus nidulans*, existing in a dispersed mycelial form, demonstrates enhanced penicillin production [8]. Research by Sai jin et al. indicates that small mycelial pellets formed by *Aspergillus niger* as dispersed mycelial fragments yield higher citric acid compared to undispersed pellets [9]. Various cultivation factors, including pH, inoculum, nutrients, and trace metals, also intricately affect mycelial morphology. For instance, certain *Rhizobium* species have a high probability of forming pellets at high inoculum spore concentrations (up to 3 × 10^9^ spores/L) [10], *Penicillium chrysogenum* strains require high pH values for pellet formation [11], and carbon sources play a pivotal role in *Aspergillus terreus* pellet formation [12]. Consequently, the study of fungal pellets has been predominantly limited to individual fungal species.

While previously reported fungal species growing in granules include *Penicillium* [13], *Aspergillus* [14], and *Rhizopus* [15], with *Aspergillus niger* being predominant, there is a paucity of literature on *Monascus* growing in pellets. *Monascus*, a filamentous saprophytic fungus, is renowned for its historical applications in medicine and food [16]. *Monascus* is known for its significant polyketide secondary metabolites, including pigments, Monacolin K, and citrinin. Despite extensive research into gene manipulation to eliminate citrinin production due to its nephrotoxic nature [17], few studies have explored the relationship between citrinin production and mycelial morphology. Stirring rates, for example, significantly impact *Monascus* red-pigment production, with higher rates resulting in greater pigment yield and shorter mycelial branches [18]. Moreover, nonionic surfactants have demonstrated the capacity to modulate pigment production and mycelial morphology during *Monascus* fermentation [19].

The primary objective of this study is to investigate the external factors influencing *Monascus* pellet formation, with a focus on optimizing pellet fermentation conditions. Additionally, this study aims to elucidate the relationship between pellet morphology and the secondary metabolite citrinin. The findings of this investigation provide valuable insights into the interplay between *Monascus* pellet morphology and citrinin production, laying a robust foundation for future applications and advancements in this field.

## 2. Materials and Methods

### 2.1. Strains and Media

The wild strain *M. purpureus* RP2, maintained in the laboratory on potato dextrose agar medium at 30 °C, was selected as the experimental strain. For liquid fermentation, *M. purpureus* RP2 was initially cultivated on potato dextrose agar medium at 30 °C for 4 days. Subsequently, the mycelium was subjected to two rounds of washing with 5 mL of sterile water and then filtered through sterile gauze to obtain the spore suspension. Freshly prepared spore suspensions, each containing 10^7^ spores/mL, were then inoculated into 30 mL of potato dextrose broth and adjusted to a pH of 5.5 in its natural state. The inoculated cultures were incubated at 28 °C for 5 days under vigorous shaking at 180 rpm. All fermentation experiments were conducted in 100 mL conical flasks, each containing 30 mL of sterile medium.

### 2.2. Shaker Pelletizing: Screening Factors

Following preliminary assessments, the influence of various factors on pellet formation by *Monascus* was systematically screened. These factors encompassed pH, carbon source, nitrogen source, and spore concentration. A pH of 5.5 (the standardized optimal value) was employed as the baseline for all screened factors unless otherwise specified. The parameters under investigation included media pH at five levels (pH 4.0, 6.0, 7.0, 8.0, and 10.0) and spore concentration at five levels (1.5 × 10^3^, 1.5 × 10^4^, 1.5 × 10^5^, 1.5 × 10^6^, and 1.5 × 10^7^ spores /mL). Additionally, various carbon sources, such as glucose, sucrose, mannitol, soluble starch, xylose, and citric acid (each at 15 g/L), were examined. Similarly, diverse nitrogen sources, including NH_4_Cl, peptone, yeast leavening, soybean meal, fish powder, and C_5_H_8_NNaO_4_ (each at 10 g/L), were investigated to evaluate their impact on fungal pellet formation. The pH of the media was adjusted using either 2 M NaOH or 2 M HCl.

### 2.3. Analytical Methods

The initial spore concentration was determined using a hemocytometer counter and a light microscope (Olympus CX43, Tokyo, Japan). To measure spore concentration, the spore solution was diluted 1000-fold before quantification. Mycelial morphology in submerged cultures was assessed through visual observation over a 72 h period, enabling the differentiation between mycelium and pellets. Pellet biomass was quantified utilizing the dry weight method. Specifically, a defined volume of fermentation broth was filtered through pre-weighed filter paper, and the mycelium was rinsed thrice with ultrapure water. Subsequently, the filter paper was dried to a constant weight at 60 °C to ascertain mycelium biomass (dry weight). The diameter of pellets was measured using a vernier caliper with a resolution of 0.01 mm, with the average diameter calculated from measurements of ten pellets.

### 2.4. Morphological Observation of M. purpureus Pellet

The morphology of the pellets was observed under an optical microscope. For more detailed observations, the micro-morphology of the pellets was examined using a su8020 scanning electron microscope (SEM; Hitachi, Ltd., Tokyo, Japan). After 5 days of liquid fermentation, the pellets were fixed using a 2.5% glutaraldehyde solution, followed by a dehydration process involving sequential ethanol solutions of varying concentrations. This dehydration procedure, lasting 10 min for each concentration and repeated twice, was utilized. Subsequently, the morphology of the pellets, post-gold spraying, was examined under SEM [20]. The internal mycelial structure of the pellets was observed by employing the paraffin sectioning technique. This method encompassed the fixation, staining, decolorization, re-staining, drying, and sealing of sections, culminating in the observation of pellet tissue under a microscope.

### 2.5. Measurement of Monascus Citrinin Production

To assess the citrinin content, the fermentation broth of *Monascus* was subjected to a pre-treatment procedure. One milliliter of the fermentation broth was mixed with 2 mL of chromatographic-grade methanol and then subjected to ultrasonic extraction, away from light, for 30 min. Subsequently, the mixture was placed in a water bath at 60 °C for 1 h and then allowed to cool to room temperature. The supernatant was obtained by centrifugation for 15 min and subsequently filtered through a 0.22 μm filter. Citrinin content was determined via high-performance liquid chromatography-mass spectrometry (HPLC/MS) under specified conditions. The analysis was conducted on an Agilent 1200 series HPLC system (Agilent, Santa Clara, CA, USA) coupled with a triple quadrupole mass spectrometer (Agilent 6460 system). The analytical column employed was an Agilent eclipse plus C18 (2.1 mm × 50 mm × 3.5 μm). Conditions for the analysis encompassed a mobile phase consisting of a 70:30 (*v/v*) mixture of 0.1% formic acid and acetonitrile, a flow rate of 0.4 mL/min, a detection temperature of 40 °C, an injection volume of 1 μL, electrospray ionization source (ESI) in positive ion mode, a spraying voltage of 3000 V, auxiliary gas at 200 °C, sphingoid gas flow rate at 11 mL/min, and sphingoid gas temperature at 325 °C. The analysis was conducted in multiple reaction monitoring (MRM) mode, and the characteristic ion pair for citrinin was 233/251.2.

### 2.6. Statistical Analysis

All experiments were performed in triplicate, and numerical data are expressed as mean ± standard deviation. Statistical analysis was conducted using GraphPad Prism 9.0, employing analysis of variance (ANOVA). Statistically significant differences were identified with *p*-values < 0.05 and <0.01.

## 3. Results

### 3.1. Effect of Initial pH on Pellet Formation

Interestingly, our investigation revealed robust pellet growth across a pH range of from 4.0 to 8.0, with a noticeable absence of pellet growth at an alkaline pH of 10.0. This observation can be attributed to the inherent negative surface charge typically exhibited by fungal spores, discouraging spore aggregation [21].

**Figure 2:**
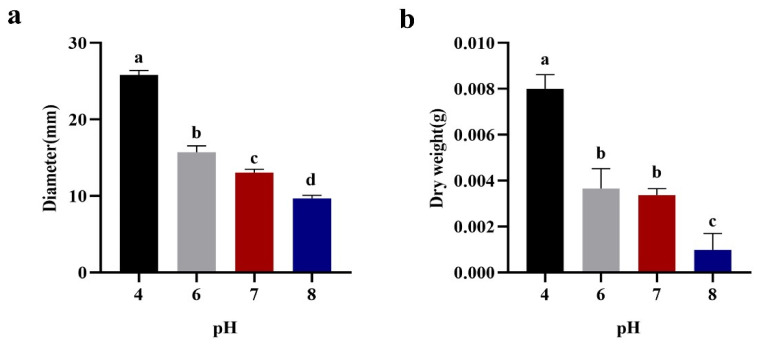
Effect of *M. purpureus* fermentation broth pH on diameter and biomass of pellets. (**a**) Diameter, (**b**) dry weight. Data labeled with different lowercase letters indicate significant differences (*p* < 0.05).

Upon closer examination, a consistent pattern emerged between pellet diameter and biomass in response to pH variations. Both parameters exhibited a reverse correlation with pH levels, as both pellet diameter and biomass decreased with increasing pH. For instance, compared to the substantial 25.64 ± 0.27 mm pellet diameter observed at an initial pH of 4.0 (Figure 2a), the pellet diameter notably reduced by 38.61%, 49.10%, and 62.24% when adjusting the medium pH to 6.0, 7.0, and 8.0, respectively. This trend was mirrored in the biomass of the pellets, as depicted in Figure 2b, where decreases of 53.75%, 56.5%, and 87.62% were observed at pH values of 6.0, 7.0, and 8.0, respectively, compared to the biomass at pH 4.0.

Remarkably, acidic conditions were found to be particularly conducive to the formation of well-defined pellets. Specifically, at pH 6.0, uniformly sized and well-segregated pellets were generated, characterized by an average diameter falling within the range from 1.4 to 1.6 mm. Conversely, at pH 4.0, although pellet size was relatively large, the pellets were not uniformly distributed, with an average diameter of 2.58 ± 0.19 mm. In contrast, at pH 8.0, pellet size was markedly smaller, with an average diameter of 0.97 ± 0.05 mm.

### 3.2. Effect of Different Carbon and Nitrogen Sources on Pellet Formation

**Figure 3:**
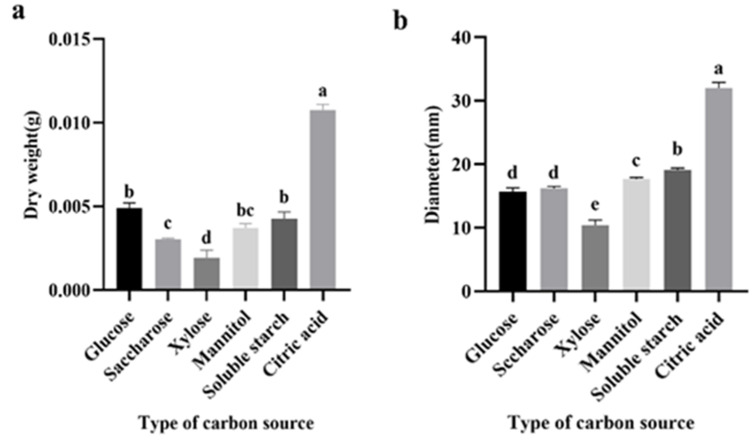
Effect of carbon source in *M. purpureus* fermentation broth on diameter and biomass of pellets. (**a**) Dry weight, (**b**) diameter. Data labeled with different lowercase letters indicate significant differences (*p* < 0.05).

In our investigation of the impact of six diverse carbon sources on pellet formation under an initial medium pH of 5.5, we observed variations in pellet formation among these sources. Notably, sucrose, xylose, mannitol, and soluble starch led to reductions of 32.10%, 56.72%, 17.17%, and 4.49%, respectively, in biomass compared to the glucose group (Figure 3a). Conversely, citric acid as the sole carbon source resulted in a 58.51% increase in biomass relative to glucose.

The choice of carbon source also influenced pellet diameter, with sucrose, mannitol, soluble starch, and citric acid contributing to increases of 2.40%, 10.51%, 17.15%, and 50.52%, respectively, in pellet diameter (Figure 3b). Xylose, however, led to a 37.48% decrease in pellet diameter. Interestingly, under carbon sources of soluble starch and xylose, a mixture of free-suspending mycelium and mycelium-forming clumps was observed.

Although citric acid yielded higher pellet biomass and diameter compared to the glucose group, visual examination revealed excessively large pellets with signs of autolysis in the central region. Consequently, our findings suggest that glucose is the preferred carbon source for optimal pellet formation.

**Figure 4:**
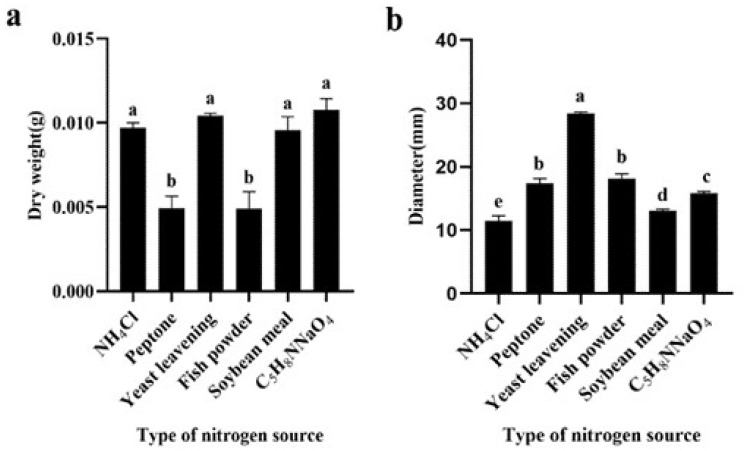
Effect of nitrogen source in *M. purpureus* fermentation broth on diameter and biomass of pellets. (**a**) Dry weight, (**b**) diameter. Data labeled with different lowercase letters indicate significant differences (*p* < 0.05).

For the effect of nitrogen sources on pellets, our study explored the impact of NH_4_Cl, peptone, yeast extract powder, soybean meal powder, fish meal, and C_5_H_8_NNaO_4_ as the sole nitrogen sources. NH_4_Cl, yeast extract powder, soybean meal powder, and C_5_H_8_NNaO_4_ significantly increased pellet biomass, resulting in respective enhancements of 49.14%, 52.17%, 48.39%, and 54.18% relative to peptone as the nitrogen source (Figure 4a). In terms of pellet diameter, NH_4_Cl, soybean meal powder, and C_5_H_8_NNaO_4_ decreased pellet diameter by 33.40%, 24.65%, and 8.84%, respectively, compared to peptone (Figure 4b). Yeast extract powder, as the sole nitrogen source, resulted in mycelium with uneven density distribution.

### 3.3. Effect of Inoculum Volume on Pellet Formation

**Figure 5:**
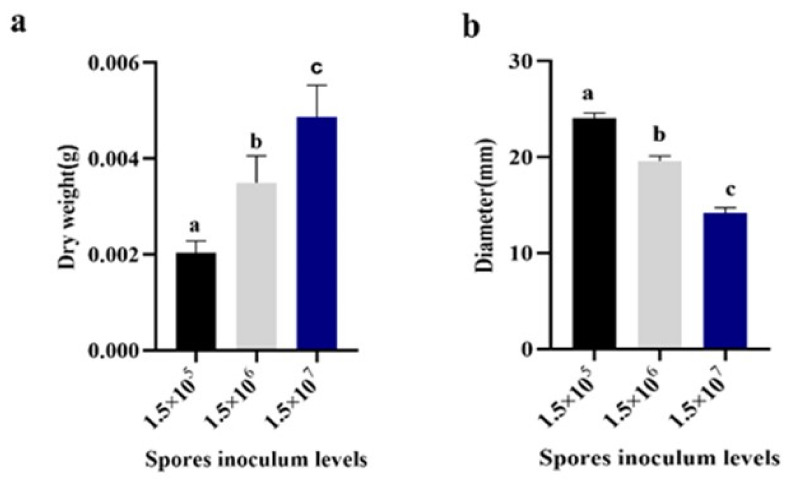
Effect of *M. purpureus* spore addition on diameter and biomass of pellets. (**a**) Dry weight, (**b**) diameter. Data labeled with different lowercase letters indicate significant differences (*p* < 0.05).

In our study, inoculum volumes were set at 1.5 × 10^3^, 1.5 × 10^4^, 1.5 × 10^5^, 1.5 × 10^6^, and 1.5 × 10^7^ spores/mL. Minimal pellet formation was observed at the 1.5 × 10^3^ and 1.5 × 10^4^ spores/mL levels. However, increasing the inoculum size led to elevated *Monascus* biomass, while pellet diameter exhibited a negative correlation with inoculum size. Relative to the 1.5 × 10^5^ spores/mL concentration, biomass increased by 1.72-fold and 2.39-fold at the 1.5 × 10^6^ and 1.5 × 10^7^ spores/mL levels (Figure 5a), respectively, while pellet diameter decreased by 18.6% and 41.06% under these respective conditions (Figure 5b). Pellets tended to form at inoculum levels below 10^8^ spores/mL, with higher concentrations favoring dispersed mycelial growth [22].

### 3.4. Relationship between Pellet Size and Citrinin

**Figure 6:**
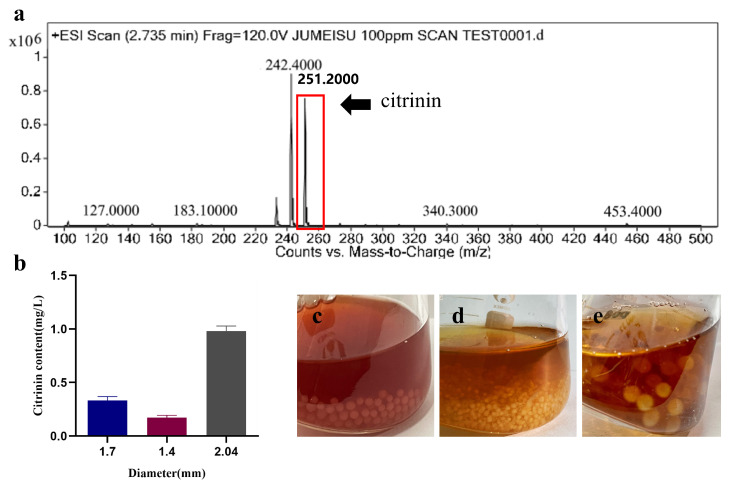
Determination of citrinin in the fermentation broth of *M. purpureus*. (**a**) Total ion flow diagram in fermentation broths, (**b**) content of citrinin in different pellet diameters. (**c**–**e**) Pellets of different diameters, (**c**) the average diameter of 1.7 ± 0.08 mm. (**d**) The average diameter of 1.4 ± 0.07 mm. (**e**) The average diameter of 2.04 ± 0.008 mm.

During the fermentation process, the intricate relationship between the production of secondary metabolites and the morphology of filamentous fungi becomes evident [23]. Varied pellet diameters seem to exert distinct influences on citrinin synthesis, with larger pellet diameters (average diameter of 2.04 ± 0.008 mm) correlating with higher citrinin content, while smaller pellet diameters (average diameter of 1.4 ± 0.07 mm) are associated with lower citrinin levels in the fermentation. This correlation is visually represented in Figure 6b, showcasing the differences in citrinin content across various pellet diameters, ranging from a few hundred micrometers to one mm (Figure 6c–e).

The internal structure of fungal pellets is characterized by a dense kernel of tightly packed hyphae [24]. While the conventional method for pellet analysis involves microscopic examination [25], this approach has limitations, including sample squeezing that can impact biomass size and the loss of three-dimensional pellet information. To comprehensively understand pellet morphology, we employed scanning electron microscopy (SEM) for both overall and internal pellet visualization, supplemented by paraffin sections to observe the internal mycelial structure.

**Figure 7:**
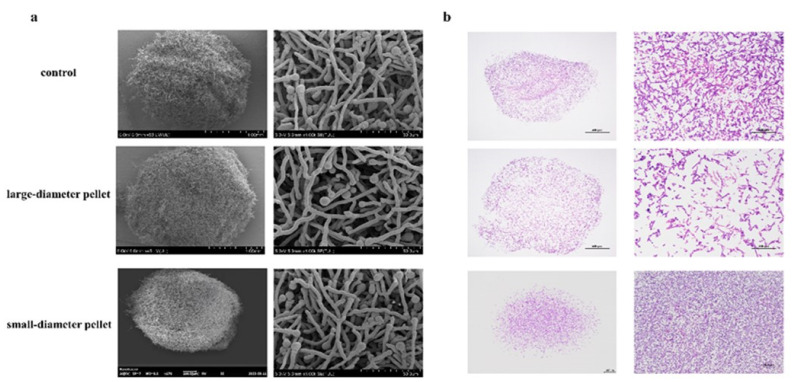
Comparison of the morphology of pellets of different diameter sizes. (**a**) Scanning electron microscope observation of the microstructure of pellets at ×170 (**left**) and ×1000 (**right**). (**b**) Paraffin sections were observed under a light microscope at×40 (**left**) and ×200 (**right**).

SEM images at 170× and 1000× magnifications (Figure 7a) unveiled the formation of pellets through the interweaving of mycelium, creating gaps between the mycelial elements. In comparison, control pellets with a mean diameter of 1.7 ± 0.08 mm exhibited a looser surface, while smaller-diameter pellets were tightly ensconced by mycelium. Notably, the mycelium structure within larger pellets appeared relatively less dense than their smaller counterparts, facilitating the expulsion of metabolites.

For a more detailed examination of the internal mycelial structure, paraffin sections were prepared for mycelial pellets cultured for 168 h (Figure 7b). Optical microscopy at 40× and 200× magnifications revealed robust overall growth and a uniform, dense distribution of mycelium within smaller pellets (average diameter of 1.4 ± 0.07 mm). In contrast, larger pellets (average diameter of 2.04 ± 0.008 mm) displayed sparse mycelium that appeared lighter in color following staining. This detailed analysis provides valuable insights into the complex interplay between pellet morphology and citrinin production.

## 4. Discussion

Industrial fermentation processes encounter challenges with filamentous fungi, and the adoption of a pelletized growth form may present solutions to some of these issues [26]. While prior studies have explored various filamentous fungal species like *Rhizopus* [10], *Aspergillus* [27], and *Penicillium* [13], all demonstrating pellet growth, it was surprising that, before this investigation, there were no reports of *Monascus* exhibiting pellet morphology. In our study, we successfully induced *Monascus* to adopt pellet form by intentionally manipulating four key factors: pH (ranging from 4.0 to 10.0), carbon source selection (including glucose, sucrose, mannitol, soluble starch, xylose, and citric acid), nitrogen source variation (NH_4_Cl, peptone, yeast leavening, soybean meal, fish powder, and C_5_H_8_NNaO_4_), and spore concentration (ranging from 1.5 × 10^3^ to 1.5 × 10^7^ spores/mL). The morphological transformations in the pellets were closely monitored over a 168 h fermentation period. Furthermore, our investigation explored the correlation between citrinin production and pellet characteristics, revealing a noteworthy observation: pellets with smaller diameters exhibited lower citrinin levels.

In our study, we systematically examined the impact of various factors on *Monascus* pellet formation, with a particular focus on pH and inoculum size. The role of pH in shaping pellet morphology is well-established, although its effects can vary among different fungal strains [11]. Our findings underscored the pivotal role of pH in *Monascus* pellet formation, with acidic conditions, specifically in the pH range of form 5 to 6, proving to be particularly conducive. Previous research on *Cordyceps sinensis* Cs-Hk1 and *Rhizopus oryzae* has reported similar observations, where lower pH levels (around 3.3 to 2.6) promoted the development of small, uniformly distributed pellets [15,28]. In our study, *Monascus* pellets formed under acidic conditions exhibited distinct characteristics, including small diameters, uniform and densely packed internal distributions, robust pellet integrity, and lower citrinin levels. This aligns with the general trend observed in fungal spores, which are negatively charged and exhibit variations in surface charges and isoelectric points [21]. The negative charge of fungal spores, expressed through electrophoretic mobility or zeta potential, increases with higher pH values, inhibiting spore aggregation [29,30]. This was substantiated by experimental demonstrations showing that increased pH hindered spore adherence to negatively charged surfaces [31]. Additionally, pH significantly influences the hydrophobicity of proteins, particularly those with hydrophobic characteristics that strongly impact adhesion [32]. 

Conversely, higher pH levels (7 to 8) in our study led to reduced biomass production and uneven pellet distribution. This observation in *Monascus* aligns with the notion that, in general, an increase in pH impedes spore aggregation due to the heightened negative charge on the conidial cell wall surface. The robust association we observed between initial pH levels and well-defined pellet formation in *Monascus*, particularly favoring acidic conditions, adds valuable insights to the understanding of fungal pelletization dynamics. Our investigation highlights the intricate relationship between initial pH conditions and *Monascus* pellet morphology, emphasizing the favorable conditions for pellet development under acidic pH values (5–6).

In shaping the morphology of filamentous fermentation, critical factors such as the quantity, type, and age of the inoculum play pivotal roles. Our study focused on elucidating the impact of spore concentration, revealing noteworthy insights into *Monascus* pellet formation. Lower spore concentrations, specifically at 1.5 × 10^7^ spores/mL, were associated with the development of small, uniformly distributed pellets. These findings align with the observed inverse relationship between spore quantity and pellet size in various *Aspergillus* species [33]. Similar trends, indicating a decrease in spore inoculum leading to the formation of larger pellets, were documented in the study conducted by Posch and Herwig [34]. Notably, there exists a discrepancy in the literature regarding the preferred inoculum concentrations for optimal pellet formation [35].

To reconcile these disparities, our study integrated insights from the morphological development of *Aspergillus niger* with spore inoculum analyses [36]. The observed trends may be attributed to the interplay between NH^4+^ uptake, glucosamine concentration, and dissolved oxygen levels. Higher inoculum concentrations appear to enhance mycelial aggregation through an accelerated NH^4+^-uptake rate, subsequently leading to the faster conversion and release of glucosamine. This released glucosamine acts as a cohesive substance, promoting mycelial aggregation and pellet formation. As the inoculum concentration continued to increase, we observed a reduction in pellet diameter, accompanied by significant changes in roughness and densification. Assigning specific factors responsible for mycelial aggregation and disaggregation proves challenging, as discussed in the review by Braun et al. [37]. Our results underscore the significance of the inoculum amount in determining mycelial morphology by influencing the internal environment of the fermentation broth and the overall fermentation rate.

*Monascus*, like other filamentous fungi, relies on organic carbon and nitrogen sources to support its growth, providing essential energy for fungal development. Our investigation explored the impact of various carbon and nitrogen sources on pellet formation, revealing diverse effects on *Monascus* morphology. Organic compounds crucial for fungal growth, including sugars like D-glucose, D-fructose, and D-sucrose, are readily assimilated and utilized by *Monascus*. Additionally, *Monascus* exhibits the capacity to utilize a spectrum of other compounds, such as polysaccharides, organic acids, amino acids, alcohols, and hydrocarbons [38]. In our experiment, different carbon sources, excluding citric acid, did not significantly decrease pellet size but influenced both biomass and pellet dimensions. All studied carbon sources yielded pellet sizes ranging from 1 mm to 3 mm, showcasing well-developed mycelium on the pellet surface, and stable-sized pellets were consistently observed. Notably, the medium supplemented with glucose as the carbon source exhibited the highest biomass, consistent with findings from other studies highlighting glucose as a preferred carbon source for biomass production [39]. Mycelia, recognized as pivotal sites for growth and branching [40], play a crucial role in the expansion of pellets. While the exact mechanism behind the influence of glucose on pellet size and biomass remains unclear, it is evident that glucose contributes to increased pellet size by directly providing energy and carbon.

Changes in nitrogen sources led to variations in both biomass and pellet diameter. Pellet diameters for all nitrogen sources, except yeast extracts, fell within the 1 mm–3 mm range and exhibited robust development. However, NH_4_Cl and C_5_H_8_NNaO_4_ resulted in a significant decrease in pellet size, aligning with similar findings in the work of Jonsbu et al. [41]. These salts were observed to induce a growth lag, effectively controlling pellet growth. Earlier reports emphasizing the promotion of pellet formation by organic nitrogen sources due to their nutrient-rich composition supporting cell growth were corroborated in our study [42]. Our investigation highlighted the profound impact of carbon- and nitrogen-source selection on *Monascus* pellet formation. These findings underscore the pivotal role of nutrient sources in shaping fungal morphology. Glucose emerged as the preferred carbon source for optimal pellet formation, while peptone and fish meal demonstrated favorability as nitrogen sources for promoting pelleting.

While the optimal pellet morphology for maximizing secondary metabolite production in *Monascus* remains undetermined, studies have demonstrated a positive correlation between productivity and specific features, such as short, swollen branches [43]. Generally, a less dense internal structure within pellets is associated with higher productivity, a trend observed in the highly productive lactic acid formation from the pellet morphology of *Rhizopus oryzae* [44]. Despite this, smaller pellets are often considered more compact and stable. The overall connection between pellet size and productivity underscores the importance of oxygen and substrate availability, highlighting the need for pellets with open channels for optimal productivity. In summary, the relationship between pellet morphology and productivity in *Monascus* fermentation is intricate, with attention to features like short, swollen branches and internal structure density playing a key role in secondary metabolite production.

## 5. Conclusions

In conclusion, the production of vital secondary metabolites by *Monascus*, including pigments, *γ*-aminobutyric acid, Monacolin K, and citrinin, is significantly influenced by changes in mycelial morphology. Our findings established a correlation between citrinin content and pellet diameter, indicating that larger pellets tend to exhibit higher citrinin content. This correlation may be attributed to the autolysis tendency of large-diameter pellets, leading to the elimination of metabolites due to their sparse distribution within the pellet structure.

To explore the growth of *Monascus* pellets, our study emphasized the pivotal roles of pH and inoculum size in pellet formation, highlighting the pH range from 5 to 6 as optimal for fostering pellet growth. Additionally, we uncovered a relationship between pellet diameter and citrinin content, demonstrating that smaller pellets harbor lower citrinin levels. Nevertheless, further research is imperative to unravel the underlying molecular mechanisms and key molecules that drive pellet formation in filamentous fungi.

## Data Availability

Data will be made available on request.
